# The heat-tolerance evaluation of an *Isochrysis zhangjiangensis* mutant generated by atmospheric and room temperature plasmas

**DOI:** 10.1186/s13568-019-0792-7

**Published:** 2019-05-21

**Authors:** Guangze Yuan, Xupeng Cao, Zhen Zhu, Miao Yang, Junpeng Jiang, Xuran Fan, Peichun Wu, Hongbin Lu, Jing Tian, Song Xue

**Affiliations:** 1 School of Bioengineering, Dalian Polytechnic University, Dalian, 116034 China; 2 Marine Bioengineering Group, Dalian Institute of Chemical Physics, CAS, Dalian, 116023 China; 3 University of Chinese Academy of Sciences, Beijing, 100049 China

**Keywords:** Microalgae, *Isochrysis zhangjiangensis*, Heat-tolerant, Fatty acid synthesis, Triacylglycerols

## Abstract

*Isochrysis zhangjiangensis* is widely used in the marine aquaculture as larval feed, especially for filter feeding cultures, as well as a good candidate for biofuels. However, the optimal cultivation temperature for *I. zhangjiangensis* is below 30 °C and this stain is seriously affected by high temperature, which causes the limited application during the summer. *I. zhangjiangensis* IM130005 is a strain generated by atmospheric and room temperature plasmas with relative higher growth rate and lipid production than the wide strain (WT), with the ability to tolerate several hours’ high temperature during the outdoor cultivation. Here, a detailed comparison was performed by continuous monitoring growth, chlorophyll fluorescence and fatty acid profile between IM13005 and WT under a mimic temperature shock to the summer outdoor cultivation. Based on a nearly 20% increase of total fatty acid in IM13005, which was majorly contributed by saturated or monounsaturated FAs in form of neutral lipids, within 5 h under the heat shock, the fatty acids and lipids synthesis variation were postulated as the physiological reason for the high temperature tolerance.

## Introduction

With the fast growth rate, microalgae are an important renewable biological resource integrating new energy, carbon reduction, water treatment, and health (Guedes et al. 2011). The large-scale cultivation with solar energy is considered as the most feasible way to the bulk production. However, the environmental factors, such as temperature (Ras et al. 2013), and light intensity (Singh and Singh 2015) are non-negligible for the success of the cultivation. Even under certain circumstance, cooling becomes a major obstacle before the improvement of sun light utilization. Besides the optimization of cultivation process, e.g. the enhancement of heat dissipation (Endres et al. 2016), screening and constructing heat-tolerant strain is urgent, too.

**Fig. 1:**
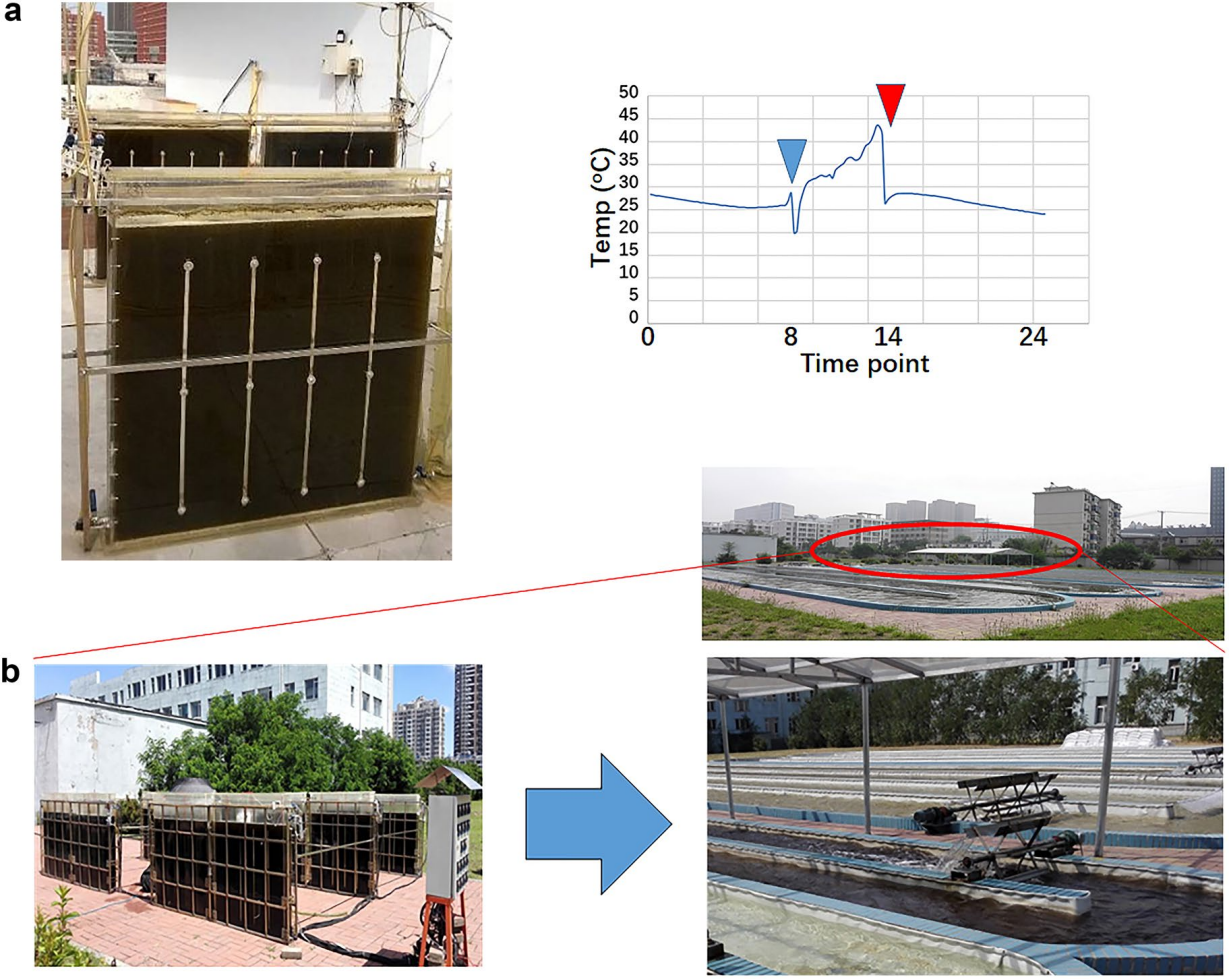
Pilot-scale cultivation of IM130005 in the summer. **a** 100 L flat bioreactor and typical temperature variation inside. The first drop of the temperature is caused by the inlet of CO_2_ enriched air and the second drop of the temperature is the result of the end of direct irradiation by the sun. **b** Using 100 L bioreactor for seeding and 1000 m^2^ race pond cultivation was achieved by two-stage amplification taken place by Shenyang Research Institute of Chemical Industry Co., Ltd

*Isochrysis zhangjiangensis* belongs to the phylum Chrysophyta and is an important larval feed for marine aquaculture (Zhu et al. 2010). It’s also a good candidate for the microalgae based biofuel R&D (Feng et al. 2011; Wang et al. 2014). The optimum growth temperature of *I. zhangjiangensis* is 25 °C and the range of 15–30 °C is adaptable for them (Guo et al. 2011). In the winter, the green house is enough to keep the temperature, but in the summer, no cheap way to cool them down. So, it’s hard to make a year-around cultivation, even in the north part of China. Figure 1a showed a typical temperature variation in 100 L flat bioreactors outdoor from July to September in Dalian (121°E, 39°N).

Since 2012, the authors have applied atmospheric and room temperature plasmas (ARTP) to generate mutant for higher fatty acid production and better environmental tolerance (Cao et al. 2014). Atmospheric and room temperature plasmas (ARTP) are a new plasma source technology developed in recent years (An et al. 2017). The ARTP mutation system induces diverse breakages in plasmid DNA and oligonucleotides and can be controlled at room temperature (An et al. 2017; Cao et al. 2014). Finally, nearly 1000 mutants of *I. zhangjiangensis* were obtained, and among them, stain IM130005 (IM) showed a better growth and heat tolerance than the wild strain (WT). Based on its good performance, increasing trials of this strain in the southeast of China have been carried out.

However, due to the randomness mutation on genome caused by ARTP, the mechanism of improved growth and heat tolerance was unknown. The previous report showed that during the colon screening by Nile red, IM have stronger fluorescence signal than WT (Cao et al. 2014) indicated that IM might have enhanced lipid synthesis ability.

Here, a detailed inspection on the response of both IM and WT were carried out on the Algal Station (AS) system (http://www.mbpe.dicp.ac.cn/yjcg/kyjz/kyjzpage.html), by continuously monitoring the change on chlorophyll fluorescence and optical density (OD), during a stepwise temperature increase process, mimicking the change of previous outdoor cultivation. The fatty acid profile as well as lipid content changes were also monitored and compared to help the understanding of its tolerance.

## Materials and methods

### Microalgae cultivation

The wild strain used here referred to *Isochrysis zhangjiangensis* FACHB-1750, which was obtained from the Liaoning Institute of Marine Fisheries and maintained by the Freshwater Algae Culture Collection at the Institute of Hydrobiology (FACHB-collection), Chinese Academy of Sciences. Mutant *Isochrysis zhangjiangensis* IM130005 was generated by ARTP techniques (Cao et al. 2014) and deposited in China General Microbiological Culture Collection Center (CGMCC) with registration number of 11418.

The microalgae was photoautotrophically cultured at 25 ± 1 °C in the autoclaved natural seawater, supplemented with threefold modified F/2 culture medium as previous report (Feng et al. 2011) and compressed air enriched with 2% CO_2_. The cultures were inoculated in a 3-L poly(polymethyl methacrylate) (PMMA) photobioreactor (20 × 5 × 30 cm in length, width and height) with a 2-L working volume under the monitoring and control of AS. The average aeration rate was 0.4 vvm and the CO_2_ was supplemented only during illumination. The cultures were illuminated from one side with white LED (AC-TL1000, Dalian Institute of Chemical Physics) which provided an average light intensity of 100 μmol m^−2^ s^−1^ under 14 h: 10 h light: dark cycle. The in-reactor temperature was monitored and control by AS with an accuracy of 0.1 °C and the heat-tolerance experiments were carried out from 25 °C to 45 °C with an increment of 1 °C/20 min. During the cultivation, OD, pH, *F*_*v*_*/F*_*m*_ and temperature were monitored with a 20 min interval. 100 mL cultures at 25, 35 and 40 °C were sampled, centrifuged and preserved at − 80 °C for lipid analysis.

### Lipid extraction, separation and analysis

#### Lipid extraction and separation

Approximately 5 mg of freeze-dried algae were extracted in 0.95 mL of chloroform/methanol/water (1:2:0.8 by volume) with sonication for 15 min at room temperature as previous report (Meng et al. 2015). After sonication, 0.25 mL of chloroform and 0.25 mL of water were added in and the solution was vortexed. After centrifugation at 3775×*g* for 5 min, the upper phase was abandoned, the chloroform phase was collected in a glass, and the algal phase was re-extracted using the same process and collecting the chloroform phase. The combined organic phases were dried by N_2_ flow. The dried residue was re-dissolved in 1 mL of chloroform (Meng et al. 2017).

Neutral lipid (NL) was separated on untreated silica plates by thin layer chromatography (TLC) with hexane–diethyl ether–acetic acid ratio of 85:15:1 (v: v) (Meng et al. 2015). NL from the algae migrated together with triacylglycerols (TAG), diacylglycerols (DAG) and free fatty acids (FFAs), and was stained by primuline and scraped for FAME analysis. Glycolipids (GL) and phospholipids (PL) were separated on treated silica plates by TLC with acetone–toluene–water ratio of 85:15:1 (v:v) (Wang and Christoph 2011). The untreated silica plates were soaked in 0.15 M (NH_4_)_2_SO_4_ for 30 s, and then sealed in the container for at least 48 h. Before use, the plates were baked at 60 °C for 2.5 h (Meng et al. 2014). GL and PL was stained by primuline and scraped for FAME analysis.

Standard samples of the lipid standards were SQDG 16:0/18:3, PE 17:0/17:0, PG 17:0/17:0, PI 16:0/16:0, DGTS 16:0/16:0 from Avanti Polar Lipids (Alabaster, AL, USA) and MGDG 18:0/18:0 and DGDG 18:0/18:0 from Matreya LLC (Pleasant Gap, PA, USA) and TAG 51:0, 17:0/17:0/17:0 from Sigma-Aldrich, USA were used as the indicator for the separations.

### FA profile analysis

For total fatty acid, approximately 5 mg dry biomass (to analyze biomass fatty acid profile) or all separated lipids fatty acid was added to 5 mL 2% H_2_SO_4_ in methanol solution and C17:0 (Sigma-Aldrich) was added as internal standard for quantification and incubated for 1 h at 70 °C (Liu et al. 2015). NL, GL and PL from the TLC were also methyl esterification as above. After transesterification, 0.75 mL de-ionized water and 2 ml *n*-hexane were added to the vial and the top *n*-hexane layer was removed and placed into vials with a small amount of sodium sulphate for GC analysis. Fatty-acid methyl esters (FAMEs) analyses were carried out by an Agilent 6890 GC instrument was equipped with a flame-ionization detector (FID) and a DB-23 capillary column (Agilent Technologies, USA, 30 m × 0.32 mm × 0.25 µm) (Meng et al. 2017).

### Reagents

If not specified, the reagents used for cultivation and analysis were analytical reagents (AR).

### Statistical analysis

All data were presented as the averages ± standard error (SE) for three biological replicates from three batches. The significance of differences was evaluated by Student’s t-test.

## Results

### Growth of WT and IM under gradual heating

In our scale-up evaluation of bioreactor cultivation of *I. zhangjiangensis*, it’s shown that IM had a better heat tolerance (unpublished data). For example, in 500 mL column reactors, while environment temperature increased from 25 to 35–40 °C, the max specific growth rates of IM remained stable, about 0.71, while that of WT decreased from 0.66 to 0.56 (Table 1). Its continuous outdoor cultivation during the summer was also successful in 100 L flat bioreactor as well as in 1000 m^2^ race pond (Fig. 1) in Liaoning Province (38–42°N) and this mutant provides a possibility to achieve year-around cultivation of *I. zhangjiangensis*.

**Table 1:** The maximum specific growth rate of IM130005 and *Isochrysis zhangjiangensis* at 25 and 35–40 °C

	Maximum specific growth rate day^−1^ (25 °C)	Maximum specific growth rate day^−1^ (35–40 °C)
IM	0.71	0.71
WT	0.66	0.56

**Fig. 2:**
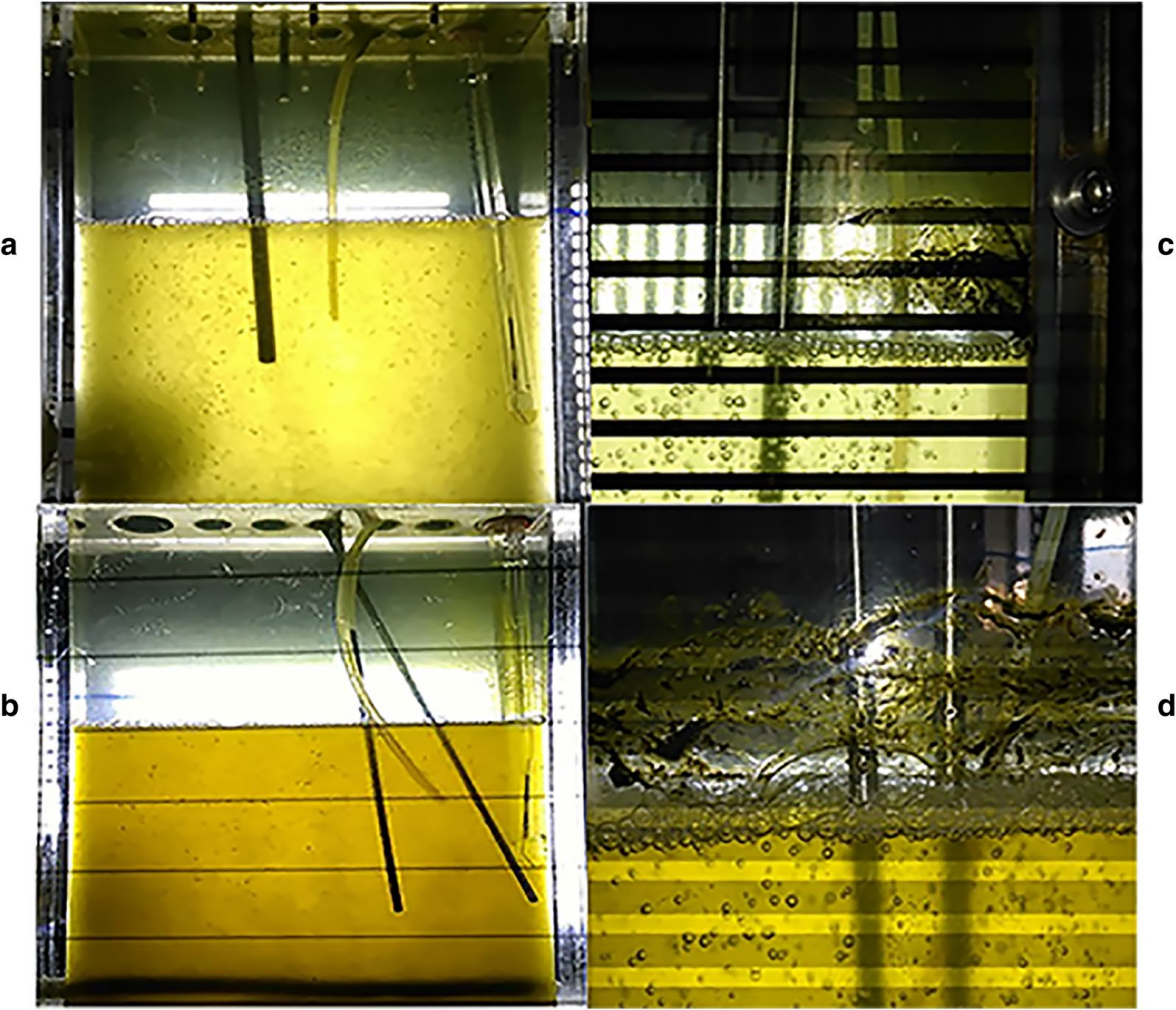
Traits of algae at different temperatures. **a** WT at 42 °C, **b** IM at 42 °C, **c** WT at 45 °C, **d** IM at 45 °C

Under the gradual heating condition, it can be clearly observed that dark green foams formed on the surface of cultures of both WT and IM while temperature increased over 42 °C (Fig. 2). However, the culture of WT bleached faster than that of IM (Fig. 2c, d). This phenomenon was the same as what happened during the outdoor cultivation of WT in the summer: the culture bleached to “clear” water within a few hours and the cultivation failed.

**Fig. 3:**
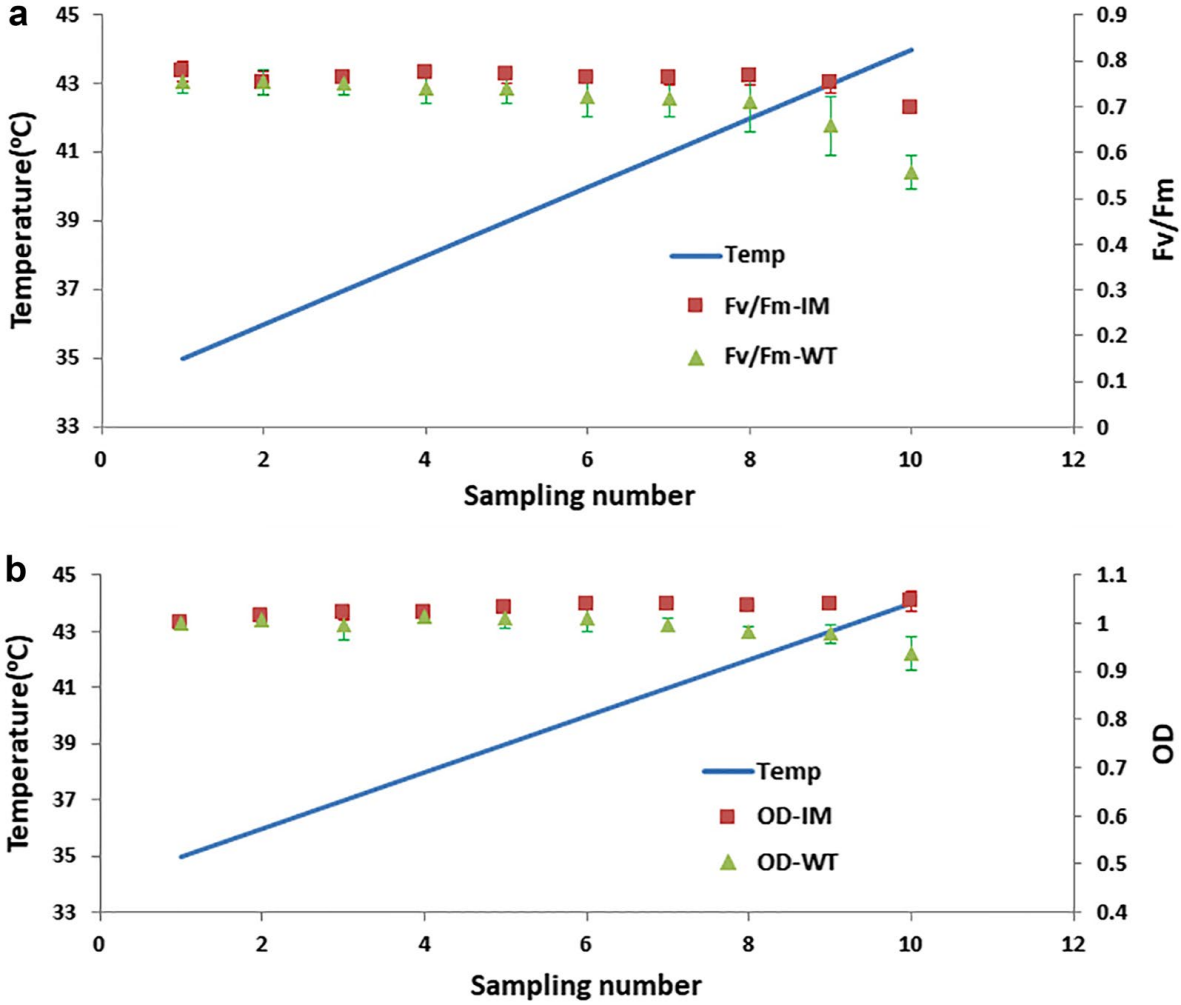
Growth and chlorophyll fluorescence curve of WT and mutant IM at different temperatures. **a** *F*_*v*_*/F*_*m*_ and **b** OD curve. All points represent the mean ± SD of three individual replicates

The continuous monitoring of OD and *F*_*v*_/*F*_*m*_ gave us dynamic information (Fig. 3). Both OD and *F*_*v*_*/F*_*m*_ were monitored and compared AS. In general, *F*_*v*_*/F*_*m*_ of both WT and IM were declined with increasing temperature over a certain temperature point. The decrease of *F*_*v*_*/F*_*m*_ happened in IM cultivation at 43 °C and remained around 0.7 at 44 °C, while in WT cultivation the turning point was 40 °C and decreased quicker than that of IM (Fig. 2a). OD showed similar trends with *F*_*v*_*/F*_*m*_ and decreased with *F*_*v*_*/F*_*m*_ (Fig. 2b). Both *F*_*v*_*/F*_*m*_ and OD changes showed IM had a better heat-tolerance than WT.

### The changes of fatty acid content (TFA) in IM and WT

**Fig. 4:**
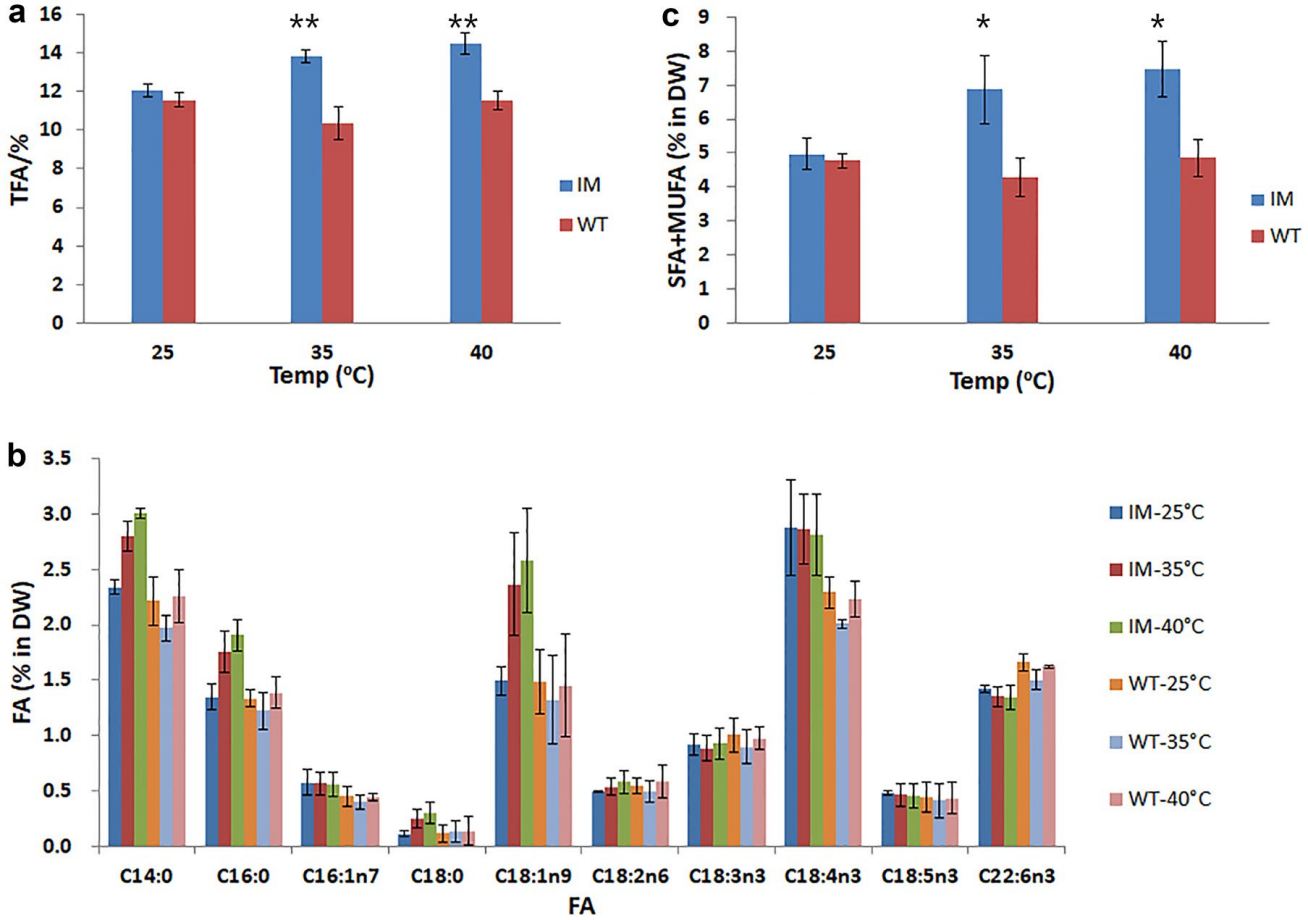
Total fatty acids and fatty acid content (% DW) of WT and IM at 25, 35 and 40 °C. **a** Total fatty acids (% DW). **b** Saturated and monounsaturated fatty acids. **c** Fatty acid content (% DW) *p < 0.05; **p < 0.01. All points represent the mean ± SD of three individual replicates

In IM, the TFA content increased from 12.0 to 14.5% as the temperature increased from 25 to 40 °C (n = 3, p < 0.05), while that of WT showed no significant difference (n = 3, p > 0.05). Lipid content of IM was 33% and 25% higher than WT at 35 and 40 °C, respectively (n = 3, p < 0.001) (Fig. 4a). Besides the content of TFA, the FA profile showed more interesting result (Fig. 4b).

It had been reported that the major fatty acid components in *I. zhangjiangensis* were myristic acid (C14:0), palmitic acid (C16:0), octadecenoic acid (C18:1n9), octadecatetraenoic acid (C18:4n3), and docosahexaenoic acid (C22:6n3). Under normal cultivation condition (25 °C), the only obvious difference in FA lied in the higher ratio of C18:4n3 in IM than in WT. In IM, responding to the heat shock, the C14:0, C16:0 and C18:1n9 content increased significantly from 25 to 40 °C (n = 3, p < 0.05), while no significant difference in C18:4n3 and C22:6n3 (n = 3, p > 0.05). In WT, only the content of C18:4n3 decreased first at 35 °C then slightly increased at 40 °C with no significant difference (n = 3, p > 0.05), while other FAs were steady (n = 3, p > 0.05).

**Fig. 5:**
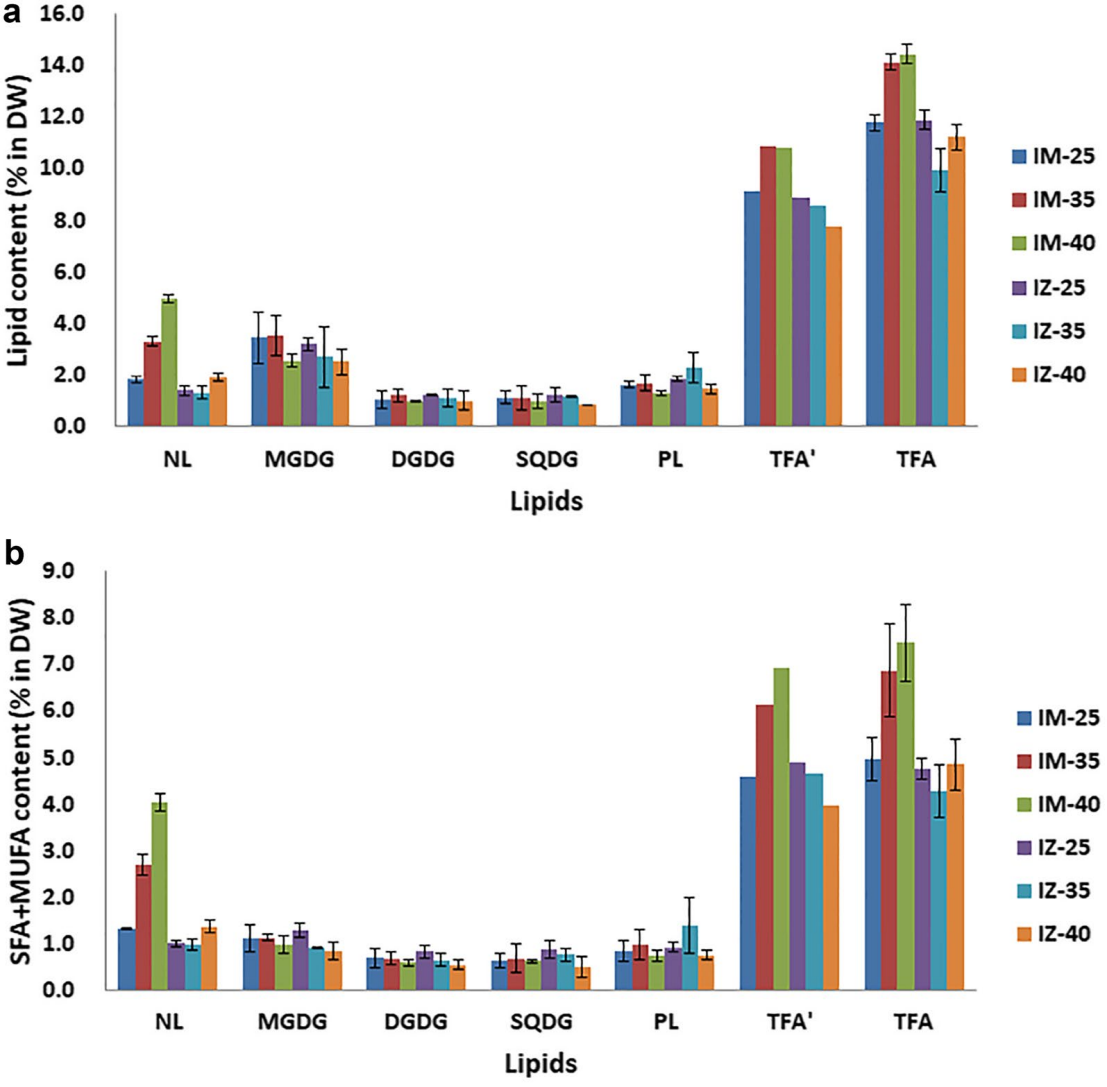
The content of total fatty acids (% DW) of WT and IM at 25, 35 and 40 °C. **a** Lipid content (% DW). **b** Saturated and monounsaturated fatty acids (% DW) of NL, GL and PL. TFA′ = NL + GL + PL. All points represent the mean ± SD of three individual replicates

**Fig. 6:**
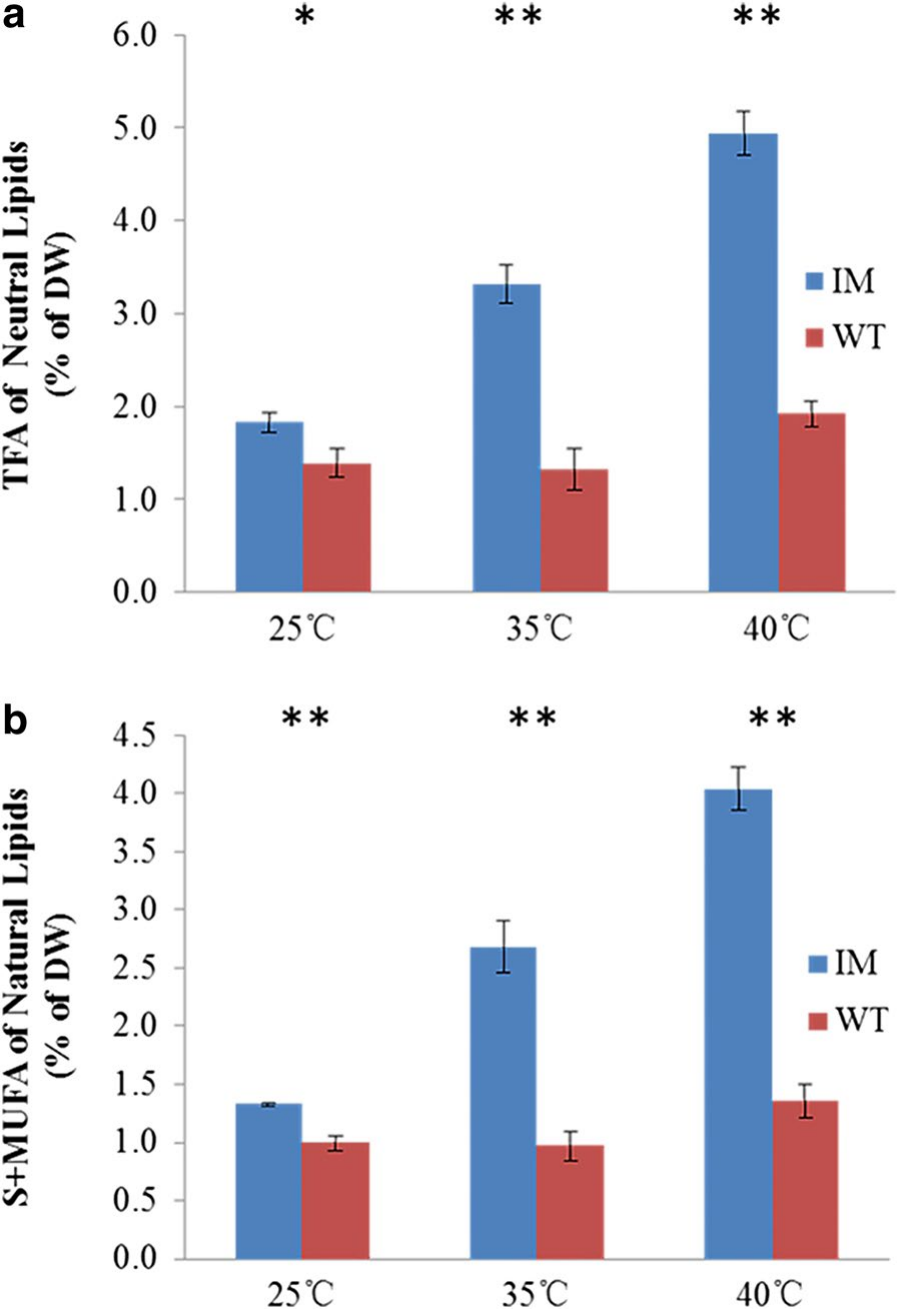
Total fatty acids of neutral lipids (% DW) of WT and IM at 25, 35 and 40 °C. **a** Total fatty acids. **b** Saturated and monounsaturated fatty acids. *p < 0.05; **p < 0.01. All points represent the mean ± SD of three individual replicates

There were no significantly difference in the saturated and monounsaturated fatty (SFA and MUFA) acids content of the mutant IM and the WT, 5.0% and 4.9% at 25 °C, respectively. However, it turned to be significant (n = 3, p < 0.05) at 40 °C (Fig. 4c) with a nearly 50% increase to 7.5% in IM while that of WT remained unchanged, 4.8%. The fast increase of SFA and MUFA resulted in a rapid accumulation of TFA in IM and the increased saturation level of lipids. For example, the ratio of SFA and MUFA increased from 48 to 60% in IM while that in WT was unchanged. Furthermore, the distribution of increased SFA and MUFA were not equally within major lipids (Figs. 5, 6).

The difference of lipid content between IM and WT is mainly reflected in NL. In IM, the NL increased significantly (n = 3, p < 0.01), about 170% from 25 to 40 °C, while of GL and PL had no significant changes (n = 3, p > 0.05) (Fig. 5a). In WT, the NL increased about 38% (n = 3, p < 0.05), while GL and PL showed no significant changes (p > 0.05) (Fig. 5a). The total NL of IM was 31% (n = 3, p < 0.05), 150% and 157% (n = 3, p < 0.01) higher than that of WT at 25, 35 and 40 °C, respectively (Fig. 6a).

**Fig. 7:**
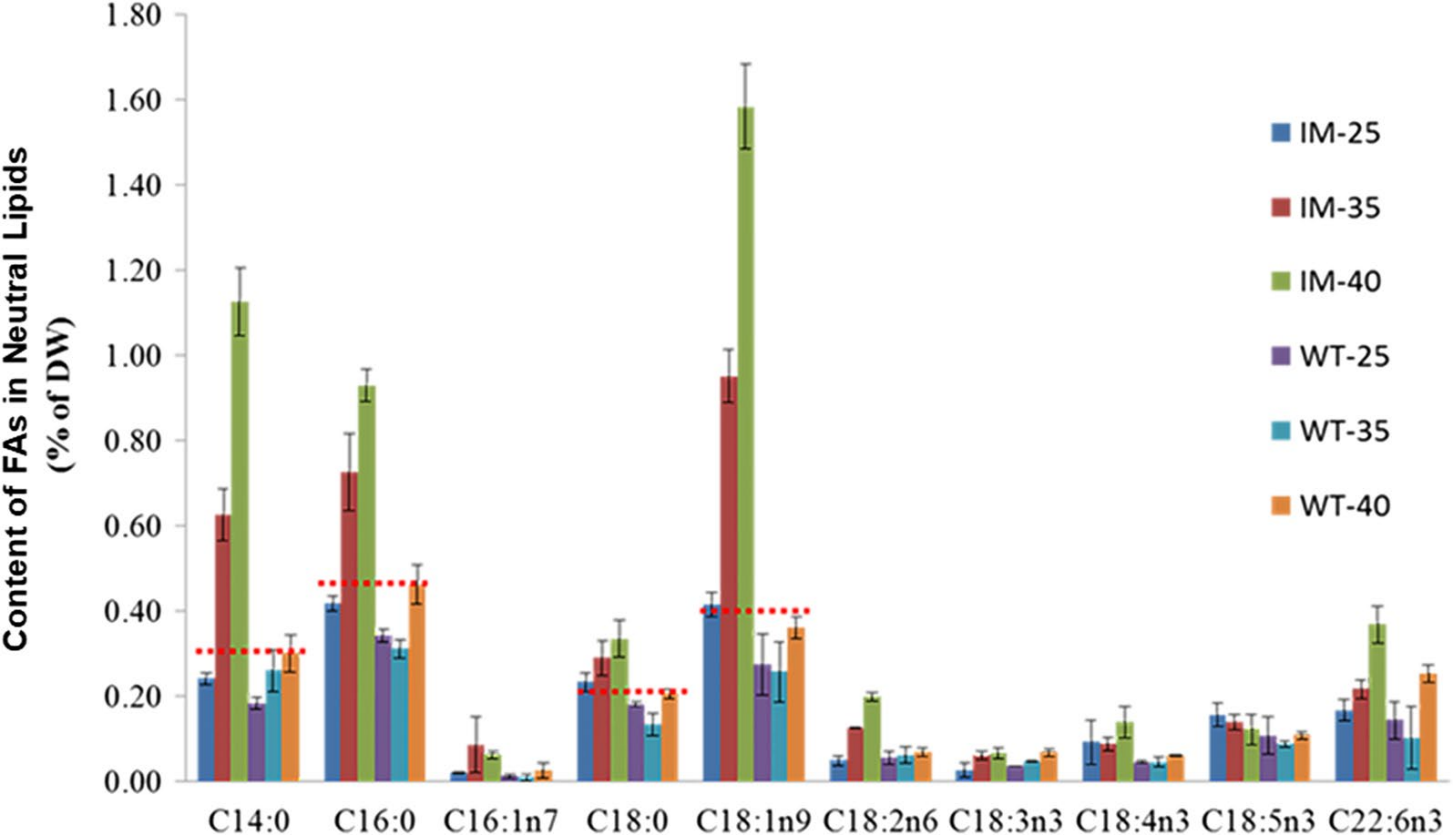
Fatty acid content (% DW) of neutral lipids of WT and IM at 25, 35 and 40 °C. All points represent the mean ± SD of three individual replicates

The SFA and MUFA in IM’s NL increased about 203%, while those in GL and PL has no significant difference (n = 3, p > 0.05) (Fig. 5b). The SFA and MUFA of NL in WT increased about 36% (n = 3, p < 0.05), and those of GL declined about 42% (n = 3, p < 0.05), while those of PL showed no significant change (n = 3, p > 0.05) (Fig. 5b). The SFA and MUFA of NL in IM was 33%, 175% and 197% higher than that of WT at 25, 35 and 40 °C (n = 3, p < 0.01), respectively (Fig. 6b). The major fatty acids changed in IM’s NL were C14:0, C16:0 and C18:1n9, with a 365, 122 and 281% (n = 3, p < 0.01) increase from 25 to 40 °C, respectively (Fig. 7).

## Discussion

In this study, the precious difference between heat tolerant strain IM130005 and WT were compared under a mimic gradual heating condition with the aid of Algal Station, from both physiology and biochemistry point of views. The quick switch of fatty acid and lipid synthesis may be the reason for IM’s improved heat tolerance, especially the quick synthesis of NL, with an average rate of 0.44% DW/h from 25 to 35 °C and doubled to 0.98% DW/h from 35 to 40 °C, while there was no significant changes in WT (Fig. 6a).

GL is the important component in photosynthesis system and in charge of the stability of the complex membrane structure of thylakoid membrane (De Los Reyes et al. 2016). C18:4n3 is the majority fatty acid in MGDG, which showed a stable higher level in IM than WT, 0.5% of DW, during the whole temperature range (Fig. 4b). It should contribute the heat tolerance by making the photosynthesis membrane more stable or by providing stronger recovery ability, which was indicated by the maintain of *F*_*v*_/*F*_*m*_ at high temperature (Fig. 3a).

Release unusable energy in form of heat is a regular energy regulation method in cells. However, under the gradual heating circumstance, it will be harder and harder. Normally, WT of *I. zhangjiangensis* accumulates carbohydrates under stress conditions, such as under nitrogen depletion (Wang et al. 2014). Other than carbohydrate, the synthesis of lipids consumes more energy. The above analysis of FA and lipids showed that IM owned a fast NL synthesis ability, in which TAG was majority. It can be postulated that the FA and lipid helped IM to branch extra energy while heat dissipation became harder in high temperature to survive. In some natural strains, heat stress stimulates the accumulation of FAs. For example in *Nannochloropsis oculata*, TFA increased from 7.90 to 14.92% when the temperature increased from 20 to 25 °C (Converti et al. 2009; Li et al. 2016). The mutation enhanced this ability in WT and IM will be a good target for understanding the fatty acid and lipid synthesis regulation in microalgae.

As a short conclusion, with the accurate control and monitoring of gradual temperature increasing cultivation by AS, and detailed analysis of FA profiles and lipid contents, the ARTP mutated strain IM130005’s good heat tolerance was inspected in physiological and biochemical aspects. The rapid switch of FA and lipid synthesis was identified as the reason for the improved heat tolerance: (1) higher level C18:4n3 in GL helps the stability of photosynthesis system under higher temperature, and (2) quicker TAG accumulation relieves the energy stress while heat dissipation is hindered under higher temperature.

## Data Availability

Please contact author for data requests.
